# Compression of the peroneal nerve by a neurofibroma originating from collaterals of the peroneal nerve: a case report

**DOI:** 10.1186/s13256-016-0815-9

**Published:** 2016-02-02

**Authors:** H. Arabi, O. Bakzaza, A. El Fikri, A. Elktaibi, H. Saidi, M. El Alaoui

**Affiliations:** 1 Equipe de recherche clinique et épidémiologique de la pathologie ostéo-articulaire, UCH Mohammed VI, Faculty of Medicine and Pharmacy, Cadi Ayyad University, Marrakech, Morocco; 2 Department of Vascular Surgery, Avicenne Military Hospital, Marrakech, Morocco; 3 UCH Mohammed VI, Faculty of Medicine and Pharmacy, Cadi Ayyad University, Marrakech, Morocco; 4 Department of Imagery, Avicenne Military Hospital, Marrakech, Morocco; 5 Department of Pathology, Avicenne Military Hospital, Marrakech, Morocco; 6 Department of Physical Medicine and Rehabilitation (PMR), Avicenne Military Hospital, Avenue de la Résistance, Gueliz, Marrakech, Morocco

**Keywords:** External popliteal sciatic nerve, Neurofibroma

## Abstract

**Background:**

Paralysis of the external popliteal sciatic nerve is a frequent pathological condition that occurs after trauma. However, etiologies other than trauma, such as tumors, are also possible. The sensory collaterals of the external popliteal sciatic nerve have a small territory of innervation at the knee, and tumors involving these nerves become symptomatic after compression of the motor nerves. We here describe the first reported case of this phenomenon.

**Case presentation:**

This case involved a lesion compressing the origin of the external popliteal sciatic nerve of a 13-year-old Moroccan boy diagnosed with a neurofibroma. He developed functional impairment of his left lower limb during a football game, and examination revealed a steppage gait. The initial diagnosis was stretching of the peroneal nerve. The definitive diagnosis of a neurofibroma was revealed by imaging and confirmed by surgery and pathology. Treatment involved total removal of the tumor; however, our patient’s steppage gait persisted.

**Conclusions:**

Our patient developed compression of the external popliteal sciatic nerve from a tumor growing on a collateral nerve. Early diagnosis is an absolute necessity in such cases. Trauma and tumors of sensory nerves can distort the diagnosis, as in this case. Ultrasound and magnetic resonance imaging can contribute to an accurate diagnosis in patients with neuropathy in the absence trauma or tomacula.

## Background

Paralysis of the external popliteal sciatic nerve (EPSN) is a frequent pathological condition that usually occurs secondary to trauma or compression of the nerve as it winds around the neck of the fibula [1, 2]. Compression of the EPSN by a neurofibroma originating from collaterals of the EPSN is rare. To the best of our knowledge, this association has never been described in the medical literature.

## Case presentation

**Fig. 1:**
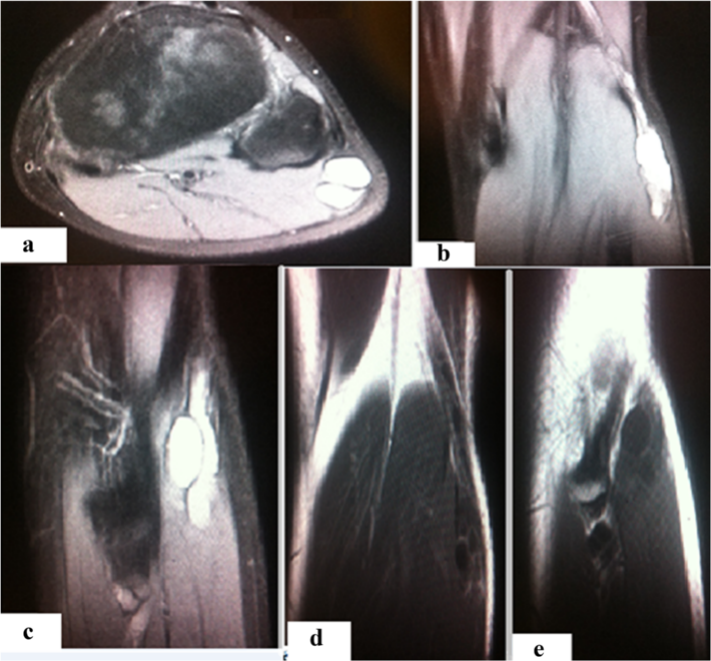
Magnetic resonance imaging. **a** Proton density fat-saturated cross-section showing a lesion projecting on to the upper outer quadrant of the patient’s popliteal fossa, a rounded area of high signal intensity and a few thin septa. **b** Proton density fat-saturated coronal section showing a lesion projecting on to the upper outer quadrant of the popliteal fossa, a heterogeneous area of high signal intensity and a few thin septa. **c** Proton density fat-saturated sagittal section showing a lesion projecting on to the upper outer quadrant of the popliteal fossa, a heterogeneous oblong area of high signal intensity and a few thin septa. **d** Coronal T1 sequence after gadolinium injection showing peripheral contrast enhancement and septa at the upper outer quadrant of the popliteal fossa. **e** Sagittal T1 sequence after gadolinium injection showing peripheral contrast enhancement and septa at the upper outer quadrant of the popliteal fossa

**Fig. 2:**
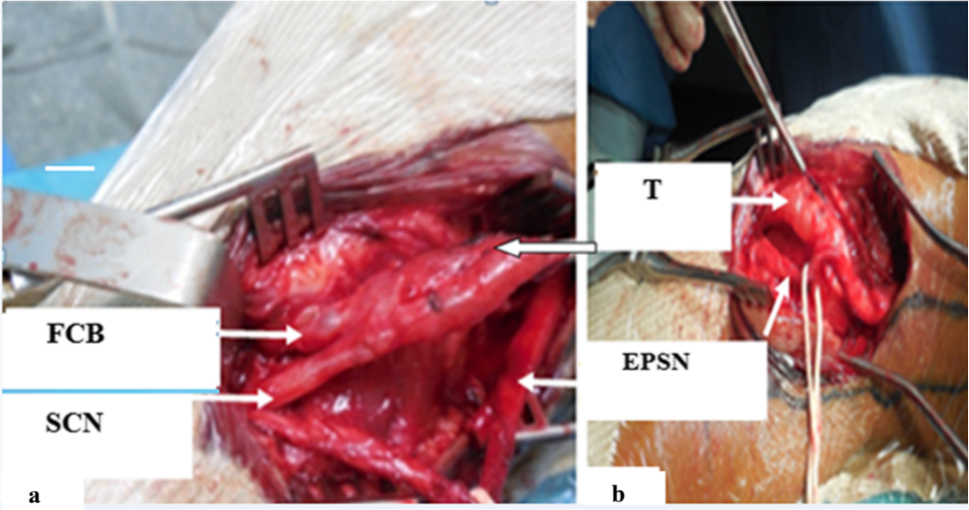
**a**, **b** Intraoperative identification of the neurofibroma over the external popliteal sciatic nerve. *EPSN* external popliteal sciatic nerve, *FCB* fibular communicating branch, *SCN* sural cutaneous nerve, *T* tumor

A 13-year-old Moroccan boy with no medical history consulted with our department regarding a functional impairment of his left lower limb that developed after a football game. No particular sprain or accident had occurred. A clinical examination revealed slight lameness without visible muscular atrophy. Muscular testing showed paralysis of his tibialis anterior and extensor hallucis longus; his common extensor digitorum was also partially deficient. Stretching of the EPSN was diagnosed based on functional disability after the football match. However, subsequent examination in our Department of Physical Medicine and Rehabilitation revealed an asymptomatic minor soft tumefaction localized at the neck of his fibula. The Tinel sign was present with a sensory deficit on the back of his foot. An electromyographic study of the nerve revealed severe damage to the EPSN at the neck of his fibula with a slow nerve conduction velocity. An electromyographic study of the nerves of his right lower limb was normal, and X-rays showed no abnormalities. Ultrasound of the tumefaction revealed a tubulated, non-compressive, anechoic mass without a Doppler signal. Magnetic resonance imaging (MRI) showed low signal intensity on T1 images, high signal intensity on T2 images, and a heterogeneous pattern due to the presence of some fine regular partitions. The lesion was in contact with the EPSN at its proximal part and coursed downward externally, following the path of its external collateral. A gadolinium injection revealed regular peripheral enhancement of the lesion, defining a central cystic area with low signal intensity (Figs. 1 and 2).

**Fig. 3:**
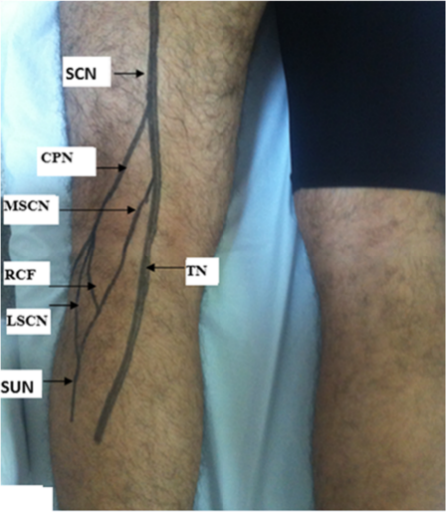
Anatomical distribution of the external popliteal sciatic nerve on the posterior aspect of the knee. *CPN* common peroneal nerve, *LSCN* lateral sural cutaneous nerve, *MSCN* medial sural cutaneous nerve, *RCF* fibular communicating branch, *ScN* sciatic nerve, *SuN* sural nerve, *TN* tibial nerve

An intraoperative examination revealed a mass at the origin of the fibular communicating branch and the lateral sural cutaneous nerves (Fig. 3). The mass was compressing but not invading the EPSN; the mass originated from collaterals of the EPSN; electrostimulation of different nerves allowed us to differentiate the motor EPSN of the sensory collateral. Finally, we completely removed the tumor with the origin of the two collateral nerves. There were no postoperative complications. A pathologic examination confirmed a neurofibroma. Our patient underwent several rehabilitation sessions, but his steppage gait persisted. Nerve grafting may eventually be conducted to address his steppage gait.

## Discussion

Compression of the EPSN frequently occurs in association with lesions at the neck of the fibula, which represents 54 % of such cases, trauma represents 40 % and a tumor origin represents only 6 % [1]. No cases of neurofibroma were mentioned in the series reported by Piton *et al*. [1]. After a review of the literature, we found only five cases of neurofibroma in relationship with EPSN, but none in relationship with its collaterals [3–5]. In the case of Mizutani *et al*., neurofibroma was found in the lumbosacral region [4]; in the case of Cebesoy *et al*., a neurofibroma involving the EPSN was a plexiform neurofibroma [5]. All cases are presented in Table 1. Ours is the first reported case of a neurofibroma of the EPSN collaterals. In fact, no tumor was present at the EPSN; instead, the EPSN was compressed by a tumor originating from collaterals of the EPSN. Trauma can distort the diagnosis, as in our case; stretching of the EPSN was first diagnosed, and this diagnosis was later corrected. The outcome of our case illustrates the importance of clinically guided diagnostic exploration, although imaging and clinical examinations remain the backbone of treatment.

**Table 1:** Reports of patients with neurofibroma of the external popliteal sciatic nerve

Authors and reference number	Year	Number of cases
Levy *et al*. [3]	1974	3
Mizutani *et al*. [4]	1989	1
Cebesoy *et al*. [5]	2007	1

A neurofibroma is a benign tumor of the connective tissue, specifically the endoneurium, of peripheral nerves [6]. It is generally associated with neurofibromatosis type I; however, it can occur in isolation, in which case it is called a solitary neurofibroma. This tumor is rare, unlike a schwannoma, which is the most frequent tumor of the peripheral nerves; the prevalence of schwannomas reached 55 % of all tumors in some series [7]. Both tumors result from the nerve sheath, which in turn originates from the neuroectoderm and neural crest. The distinction between a schwannoma and neurofibroma is important. Neurofibromas deeply invade the nerve, and total resection has functional consequences; in contrast, schwannomas arise from Schwann cells and form a macroscopically smooth, rounded, yellowish, encapsulated proliferation, the enucleation of which is easy and without loss of continuity [6]. In our patient, muscle deficiency was the first condition to appear; his EPSN was not invaded but compressed for a long period of time, and there were no sensory disturbances except for the Tinel sign. The tumor invaded the origin of both collaterals of his EPSN. Surgery involved removal of the tumor and two proximal invaded nerves with preservation of his EPSN. Only partial improvement occurred after rehabilitation. Our patient’s steppage gait persisted; nerve grafting might eventually be performed.

No neurofibromas of the collaterals of the EPSN in the lower limbs have been previously described in the literature. Most tumors in this region are schwannomas, which frequently develop at the posterior tibial nerve in the tarsal tunnel [8].

Anatomically, the EPSN is located in a musculoskeletal tunnel resting directly on the side of the neck of the bypassing fibula. The nerve is then divided between the origins of the peroneus longus muscle into two terminal branches: the deep peroneal nerve and the superficial peroneal nerve. The collateral branches of the common peroneal nerve are the peroneal communicating branch and the lateral sural cutaneous nerve (Fig. 3).

Axial ultrasound sections of the common peroneal nerve can show the lateral sural cutaneous nerve and sometimes the fibular communicating branch [9]. In their classic forms, peripheral nerve tumors are well-defined, round, oval or spindle hypoechoic masses [10]. However, it is difficult to differentiate between schwannomas and neurofibromas on ultrasound. Close attention must be paid during the diagnostic process [9]. Conventionally, schwannomas appear as eccentric masses with a homogeneous structure, acoustic posterior enhancement, intratumoral cystic modifications, and intratumoral vascularity on color Doppler examination. In contrast, neurofibromas appear echogenic and hypovascular on Doppler examination [11, 12]. In most cases, MRI permits differentiation between neurofibromas and schwannomas. A rounded appearance, peripheral location, and more or less homogeneous, central low signal intensity on T2-weighted images are the characteristics of schwannomas; neurofibromas are usually heterogeneous on both T1 and T2 images [13, 14].

## Conclusions

Neurofibroma is an uncommon tumor. Compression of the EPSN by growth of a tumor on its collaterals is particularly rare. Early diagnosis is an absolute necessity. Any delay is detrimental to the nerve and its function. Examination before the development of a steppage gait or muscular deficit of the anterior lateral compartment is necessary for a diagnosis. Ultrasound and MRI can contribute to an accurate diagnosis in patients with neuropathy in the absence trauma or tomacula. Surgery involves resection of the tumor; enucleation is desirable when the mass is well circumscribed.

## Consent

Written informed consent was obtained from the patient’s legal guardian(s) for publication of this case report and any accompanying images. A copy of the written consent is available for review by the Editor-in-Chief of this journal.

## Abbreviations

EPSN: external popliteal sciatic nerve
MRI: magnetic resonance imaging
